# Disseminated Miliary and Intestinal Tuberculosis Mimicking Inflammatory Bowel Disease

**DOI:** 10.7759/cureus.50002

**Published:** 2023-12-05

**Authors:** Danny Tran, Kunaal Patel, Areeba Ashfaq, Brittany Lyons

**Affiliations:** 1 Internal Medicine, Trident Medical Center, Charleston, USA

**Keywords:** pancytopenia, hbv, granuloma, ileitis, tuberculoma, intracardiac mass, mycobacterium tuberculosis, disseminated miliary tuberculosis, extrapulmonary tuberculosis, intestinal tuberculosis

## Abstract

The hematogenous dissemination of *Mycobacterium tuberculosis* (*M. tb*) is commonly via the pulmonary system. Less commonly, ingestion of *M. tb* can lead to primary intestinal tuberculosis (TB), often misdiagnosed as inflammatory bowel disease (IBD). In extremely rare cases, the dissemination can involve cardiac infiltration/tuberculoma. One such case involves a 21-year-old man from Guatemala who spoke a rare dialect of Spanish with nonspecific complaints and an abdominal CT scan showing terminal ileum thickening suggestive of Crohn’s disease (CD). A colonoscopy revealed ileitis and tissue biopsy showed granulomatous inflammation with a positive acid-fast bacillus (AFB) stain and positive blood cultures isolated for TB. Chest CT angiography (CTA) also revealed miliary nodules and a right atrial mass was confirmed with cardiac MRI. Viral serology revealed chronic hepatitis B virus (HBV) co-infection, but the patient was HIV-negative. Anti-tubercular therapy (ATT) with rifampin, isoniazid, pyrazinamide, and ethambutol (RIPE), in addition to tenofovir, was initiated, followed by a complicated hospital stay including rifampin-induced bone marrow suppression. Ultimately, he was discharged on isoniazid, pyrazinamide, ethambutol, levofloxacin, and entecavir. Intestinal TB can be misdiagnosed as IBD with the administration of steroids, potentially worsening infection. A systemic approach to clinical investigation with a thorough history using medical translators can lead to early diagnosis and treatment of intestinal and disseminated TB.

## Introduction

Tuberculosis (TB) is a disease caused by *Mycobacterium tuberculosis* (*M.tb*). Although it is commonly a pulmonary infection, TB can be a multi-systemic disease with hematogenous spread beyond the pulmonary system, commonly known as disseminated TB [1]. Dissemination can develop from the progression of the primary infection or reactivation of latent infection with hematogenous spread, especially in the setting of immunosuppression [2]. Miliary TB is a serious and deadly form of disseminated TB involving widespread tubercle bacilli causing millet seed-size lesions in imaging studies [3]. The signs and symptoms of disseminated TB are non-specific, depending on the affected organs and immune status of the patient.

Extrapulmonary TB accounts for nearly 15% of all TB infections worldwide [1]. Abdominal TB represents 2% of worldwide TB infections [4]. Finally, intestinal TB is rare in the United States (US) and can manifest very similar to the clinical and pathological manifestations of Crohn's disease (CD).

Extrapulmonary cardiac manifestations of TB are rare and commonly include pericarditis, myocarditis, and aortitis [5]. However, an intracardiac tuberculoma, a tumor-like mass is an extremely rare finding. It can cause serious complications such as significant arrhythmias, complete heart block, and acute heart failure [5]. The severity of the complications and vague symptom presentation make it vitally important to accurately diagnose in a systematic and timely approach.

This article was previously presented as a poster at the 2023 Trident Health Annual Research Day on May 11, 2023.

## Case presentation

**Figure 1:**
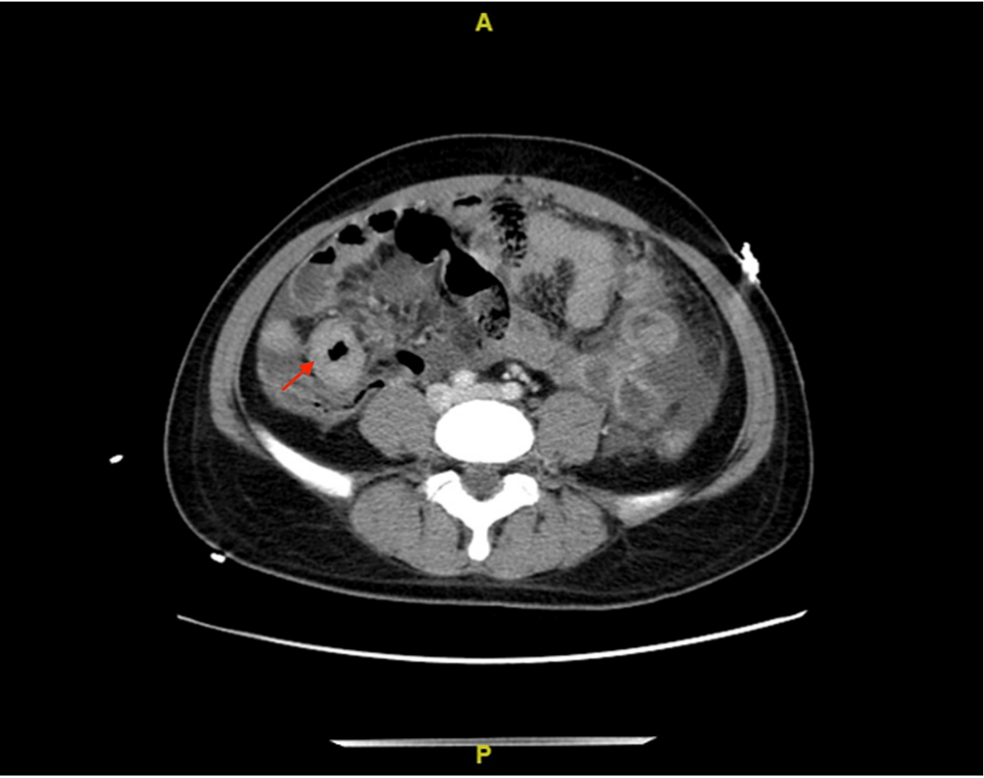
Contrast-enhanced abdomen and pelvis CT revealing full segmental thickening of terminal ileum (red arrow)

The patient was a 21-year-old Hispanic male who presented with several weeks of nausea, vomiting, and anorexia. Heart palpitations and chest pain prompted him to come to the ED. He was from Guatemala and came to the US for work. The use of an interpreter was difficult because he spoke a rare dialect of Spanish. His brother was the only person available to assist with communication between the primary team, the interpreter, and the patient. His blood pressure was 119/77 mmHg, heart rate had a regular rhythm with 129 beats per minute, respiratory rate was 18 breaths per minute, temperature was 98.4 F, and SpO2 was 95% on room air. Physical examination revealed masses with various stages of erosion on his chest wall and back. Pictures of masses were not obtained. Abdominal CT with IV contrast was significant for full segmental thickening of the terminal ileum with skip lesions and skin lesions suggestive of CD (Figure 1).

**Figure 2:**
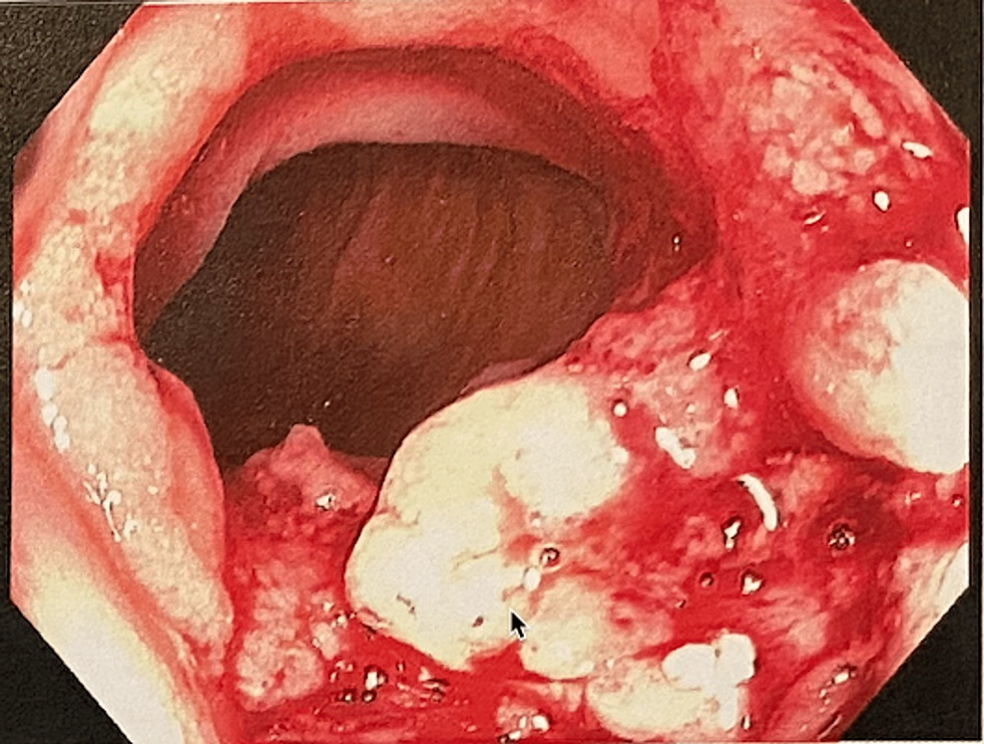
Colonoscopy findings of ileitis: severe inflammation with erythema, edema, erosions, friability, blood, pseudopolyps, and aphthous ulcers in the terminal ileum

A colonoscopy revealed severe ulcerations of the ileum with erythema, edema, erosions, friability, and spontaneous bleeding (Figure 2). Tissue biopsy showed acute ulcerative and granulomatous inflammation, consistent with CD. Of note, the pathology report did not mention if these granulomas were caseating or necrotizing. The tissue stain of the terminal ileum was positive for acid-fast bacillus (AFB).

Axillary lymph node biopsy demonstrated necrotizing granulomas consistent with TB infection but AFB stain was negative. A plan for skin lesion biopsies was stopped because the tissue AFB stain of the ileum was positive. QuantiFERON-TB Gold was also positive.

**Figure 3:**
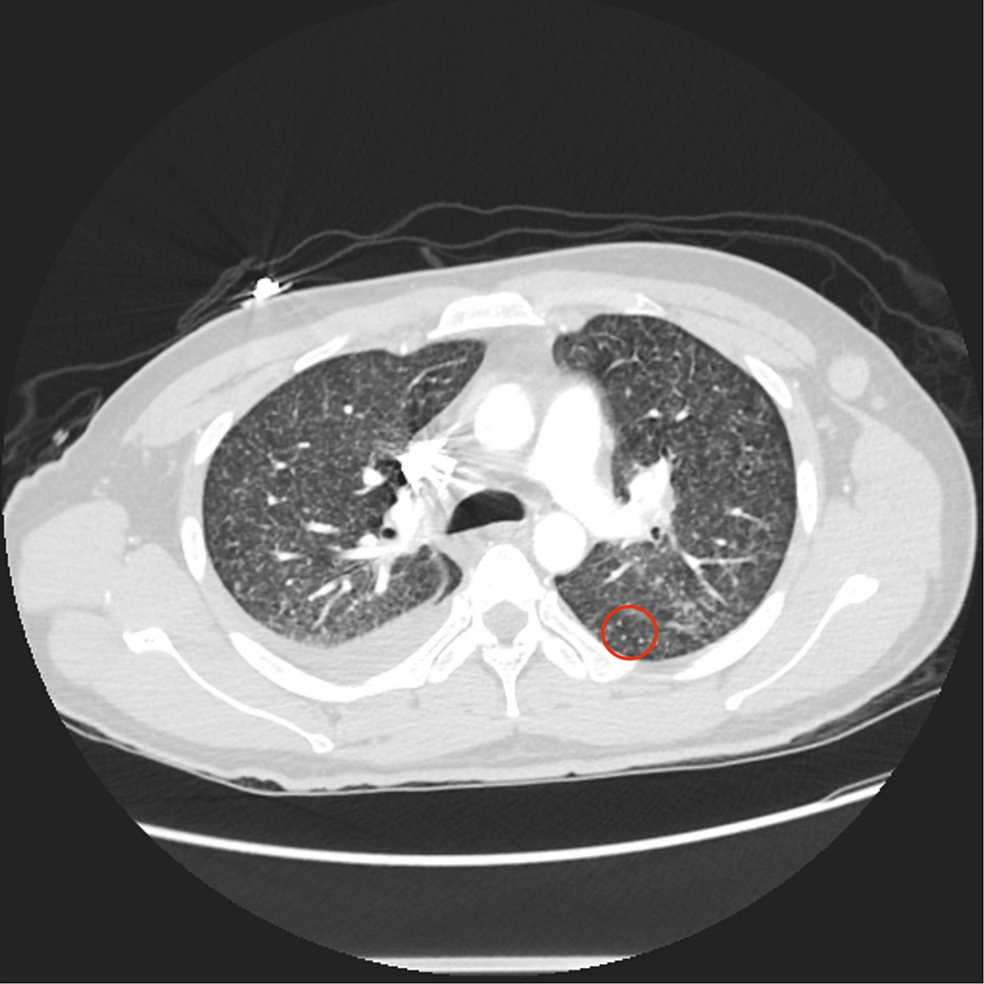
Chest CT with angiography revealing bilateral pulmonary miliary nodules (red circle)

**Figure 4:**
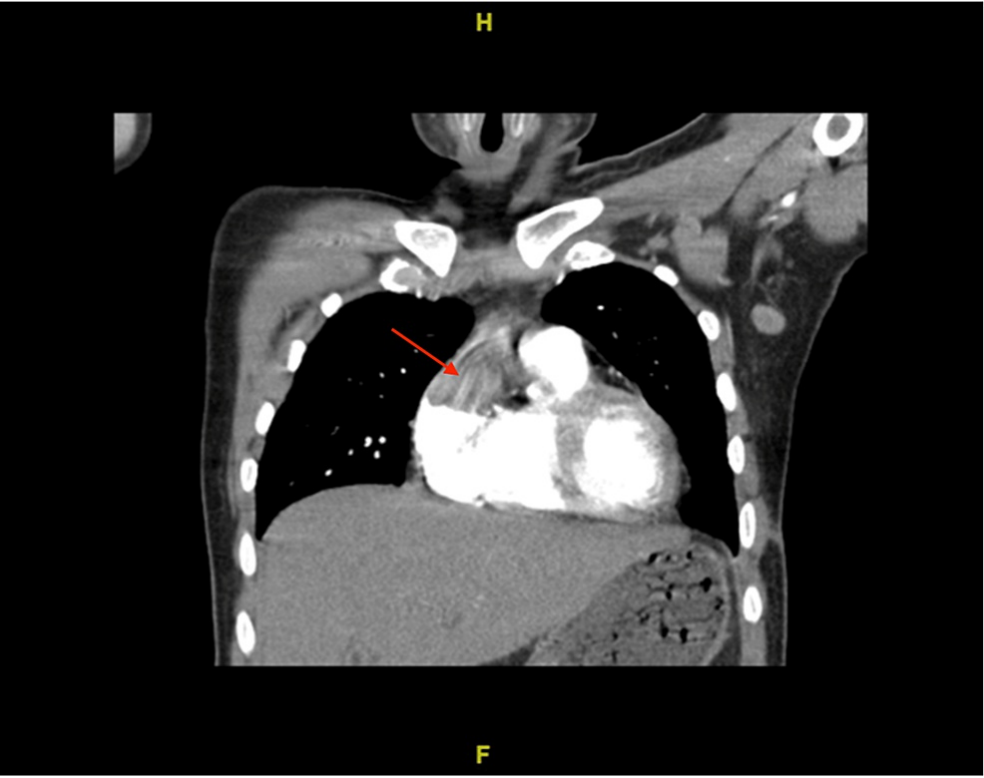
Chest CT with angiography revealing intracardiac mass in the right atrium (red arrow)

**Figure 5:**
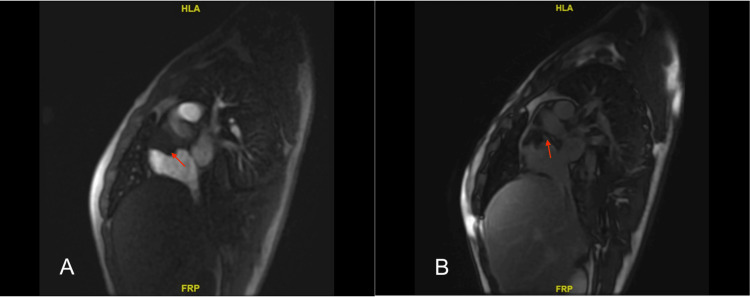
Contrast-enhanced cardiac MRI. A: Solid mass in the right atrium (red arrow); B: Late heterogeneous enhancement of mass, suggesting thrombus is less likely (red arrow)

Due to increasing dyspnea, chest CT angiography (CTA) was completed and showed miliary nodules and a right atrial intracardiac mass, which was confirmed on contrast-enhanced cardiac MRI (Figures 3, 4, 5). Respiratory cultures and AFB were negative, and it was deemed too high risk to intervene on the mass suspicious for a tuberculoma. Fungal studies were negative.

The patient was also co-infected with immune-active chronic hepatitis B. Viral serology results were positive Hepatitis B surface antigen (HBsAg), negative Hepatitis B surface antibody (anti-HBs), positive total antibody to hepatitis B core antigen (anti-HBc), negative IgM antibody to hepatitis B core antigen (IgM anti-HBc), negative hepatitis B envelope antigen (HBeAg), negative hepatitis B envelope antibody (anti-HBe). Hepatitis B viral load increased from a baseline of 750 international units per milliliter (IU/ml) on admission to 1150 IU/ml two days after initiation of rifampin, isoniazid, pyrazinamide, and ethambutol (RIPE). The combined HIV antibody and antigen test was negative. The hepatitis C antibody was negative; 600 milligram (mg) rifampin per os (PO), 300 mg isoniazid PO, 1500 mg pyrazinamide PO, 1200 mg ethambutol PO (RIPE) with 50 mg pyridoxine PO daily was empirically started due to suspected disseminated TB prior to culture data finalizing and 300 mg tenofovir disoproxil fumarate PO daily was started to treat the hepatitis B virus infection. Blood culture was subsequently positive for AFB and *M. tb* complex was isolated using a DNA polymerase chain reaction (PCR) test five weeks later.

Drug susceptibility testing revealed that the TB was sensitive to streptomycin, isoniazid, rifampin, ethambutol, and pyrazinamide. The patient’s course was complicated by severe pancytopenia with a nadir hemoglobin 5.8 g/dL, platelet <10 x 103/mm3, white blood cell 1.8 x 103/mm3, and acute liver injury (Tables 1, 2). Bone marrow tissue biopsy was normocellular with trilineage hematopoiesis and atypical megakaryocytes. Immunohistochemistry revealed no evidence of blasts. Flow cytometry demonstrated no evidence of acute leukemia, no clonal B-cell population or aberrant T-cell population, and granulocytes with a marker pattern suggesting a myeloid left shift. Peripheral smear revealed normocytic hypochromic severe anemia with moderate anisopoikilocytosis, moderate leukopenia with myeloid left shift, and severe thrombocytopenia. Bone marrow stained negative for AFB. RIPE and tenofovir were stopped due to thrombocytopenia. Treatment was changed to levofloxacin, amikacin briefly but ultimately isoniazid, pyrazinamide, and ethambutol were restarted with the cessation of rifampin. Methylprednisolone was initiated for a suspected paradoxical reaction following anti-tubercular therapy. Entecavir replaced tenofovir. IV Ig (IVIG) and platelet transfusions were started to treat rifampin-induced thrombocytopenia. These treatment changes led to significant improvement in his cell lines on the day of discharge (Table 1). 

**Table 1:** Complete blood count revealing rifampin-induced thrombocytopenia MCV = mean corpuscular volume; MCH = mean corpuscular hemoglobin; MCHC = mean corpuscular hemoglobin concentration; RDWSD = erythrocyte distribution width standard deviation; RDWCV = erythrocyte distribution width coefficient of variation

Lab Test	Day 1	Day 10	Day 26	Day 43	Day 53	Day 74	Reference Range
	Admission	Rifampin started	Rifampin stopped	Rifampin restarted	Rifampin stopped	Discharge	
WBC	8.4	6.3	3.8	5.2	3.6	5.3	4.0-10.9 10^3^/mm^3^
RBC	5.28	5.01	3.34	3.28	2.96	3.73	4.10-5.60 10^3^/mm^3^
Hemoglobin	11.8	11.1	7.5	9.0	8.0	11.5	13.5-16.5 g/dL
Hematocrit	38.1	36.3	24.0	27.6	24.6	37.1	39.0-50.0 %
MCV	72.2	72.5	71.9	84.1	83.1	99.5	80.0-95.0 fL
MCH	22.3	22.2	22.5	27.4	27	30.8	26.0-32.0 pg
MCHC	31.0	30.6	31.3	32.6	32.5	31.0	32.0-35.0 g/dL
RDWSD	39.7	41.8	50.5	60.4	59.6	68.1	35-46 fL
RDWCV	15.3	17.2	19.9	19.8	19.4	18.5	11.5-15.0 %
Platelet	291	219	120	< 10	36	120	135-350 10^3^/mm^3^

**Table 2:** Blood chemistry panel revealing acute liver injury BUN = blood urea nitrogen; GFR = glomerular filtration rate; MDRD = modification of diet in renal disease; CrCl = creatinine clearance; CG = Cockgraft-Gault; IBW = ideal body weight; ABW = actual body weight; AST = aspartate aminotransferase; ALT = alanine aminotransferase; ALP = alkaline phosphatase; CRP = C-reactive protein

Lab Test	Day 1	Day 10	Day 26	Day 43	Day 53	Day 74	Reference Range
	Admission	Rifampin started	Rifampin stopped	Rifampin started	Rifampin stopped	Discharge	
BUN	9	7	9	17	14	17	6-20 mg/dL
Creatinine	0.7	0.6	0.6	0.4	0.3	0.3	0.7-1.2 mg/dL
GFR by MDRD	> 60	> 60	> 60	> 60	> 60	> 60	>=60
Estimated CrCl CG/IBW	123.0	144.0	144.0	216.0	288	285	>60 mL/min
Estimated CrCl CG/ABW	150.3	175.3	153.7	204.9	350.6	270.4	>60 mL/min
Total bilirubin	0.7	0.7	6.4	7.1	7.9	1.3	<1.0 mg/dL
AST	32	139	129	112	88	55	<35 units/L
ALT	26	65	92	105	64	92	10-63 units/L
ALP	228	245	207	512	415	224	32-101 units/L
CRP	11.7						0.00-1.00 mg/dL

The patient was discharged home on entecavir, prednisone, isoniazid, pyrazinamide, ethambutol, and levofloxacin. The anti-tubercular medications were managed by the health department. The patient had follow-up appointments with an infectious disease clinic and cardiothoracic surgery for repeat imaging of the intracardiac mass. Communication regarding his plan, disease process, and symptoms relied heavily on the patient’s brother due to the patient’s specific dialect of Spanish.

## Discussion

TB should be highly suspected when a patient comes from a TB-endemic country complaining of cough or constitutional symptoms. This would prevent a lapse in diagnosis and treatment and prevent public and healthcare worker exposure to TB.

The clinical presentation of CD and intestinal TB both share vague and non-specific symptoms such as abdominal pain, diarrhea, and weight loss. Intestinal TB usually progresses slowly and insidiously but can present acutely with complications such as obstruction, perforation, and fistula formation [6]. The most common complication is obstruction from stricture formation [7]. According to a recent review by Kedia et al., diarrhea, hematochezia, and perianal disease favored the diagnosis of CD, while fever, night sweats, lung involvement, and ascites favored the diagnosis of intestinal TB [8]. Presentation of CD and TB lesions on both CT scan and colonoscopy may look very similar; however, in intestinal TB, right colon, cecum, and ileocecal valve involvement is more common, in contrast to left colon and terminal ileum involvement in CD [7,9,10]. In regard to endoscopic characteristics, transverse ulcers are generally found in intestinal TB while longitudinal ulcers and aphthous ulcers with skip lesions are distinctive of CD [8]. Interestingly, our patient had skip lesions. Both diseases share characteristic histological findings of granulomas. However, granulomas in intestinal TB are caseating with a central region of necrosis [3]. Lymphoma, *Yersinia enterocolitica* (*Y. enterocolitica*) infection, and certain fungal and parasitic infections can also present similarly and must be part of the differential [11]. The patient’s clinical presentation was not clear for CD since he did not have diarrhea. We subsequently collected a QuantiFeron-TB gold test, which was positive, and disseminated TB was confirmed with a positive ileal tissue AFB smear. 

Our team did a full evaluation for TB and other co-infections, despite evidence of possible CD. Initiating an immunosuppressant could have reactivated the patient’s chronic Hepatitis B (CHB) or worsened the dissemination of his tuberculosis. We assessed for reactivation with a viral load when he developed transaminitis and hyperbilirubinemia. The infectious disease team started treatment because the viral load and transaminitis were rising. Current CHB treatment guidelines recommend treating immune-active HBeAg-negative CHB when viral load is > 2000 IU/ml and there is evidence of significant histologic disease or when ALT is ≥ 2 upper limit of normal (ULN). Viral load cutoff is not an absolute requirement for treatment [12].

Primarily only a cholestatic pattern of liver injury initially presented on admission in the setting of normal transaminase (ALT, AST) levels, predominantly elevated alkaline phosphatase (ALP) levels, and R-value < 2 (0.18). Current guidelines categorize hepatocellular etiology with predominantly transaminase elevation, cholestatic with predominantly ALP elevation, or mixed. R-value (serum ALT/ULN divided by serum ALP/ULN) helps to define this more specifically. An R-value > 5 identifies cases of hepatocellular liver injury, whereas an R-value < 2 categorizes cases of cholestatic liver injury, and an R-value between 2 and 5 reflects a mixed liver injury pattern [13]. Gamma-glutamyl transferase (GTT) level was elevated at 107 mg/dL supporting a hepatobiliary source of ALP. Common causes of isolated ALP elevation are chronic cholestatic diseases, infiltrative diseases, or drugs [13,14]. The suspected causes were intrahepatic etiologies of cholestasis such as the patient’s infiltrative granulomatous disease of disseminated TB or intrahepatic fibrosis from CHB infection. The patient did not have recent exposure to offending drugs prior to admission. CT abdomen and pelvis with IV contrast did not reveal hepatobiliary pathology. A right upper quadrant abdominal ultrasound revealed mildly decreased echogenicity of the liver but did not reveal biliary duct dilatation supporting an intrahepatic etiology. Antimitochondrial antibody (AMA) was negative. Alpha-fetoprotein (AFP) level was normal. Magnetic resonance cholangiopancreatography (MRCP) was not ordered.

On hospital day 10, approximately before TB treatment was started, the patient’s transaminase levels were higher than normal. AST, ALT, and ALP levels continued to rise and total bilirubin levels became significantly higher than normal after treatment. Inconsistent and few direct bilirubin levels were obtained, so the data points were not reliable. Multiple possible factors were suspected to cause this pattern of liver injury transformation including drug effect, reactivation of HBV, and TB involvement of the liver. The R-value increased to 0.72 and indicated persistent and predominantly cholestatic injury; however, a new hepatocellular injury developed before TB treatment was initiated. The infectious disease team thought that the timing was unusual for drug-induced liver injury (DILI). DILI from isoniazid and pyrazinamide usually occurs after a few weeks and rifampin was unusual after two doses. Therefore, the new hepatocellular injury and persistent cholestatic injury with hyperbilirubinemia were suspected to be from a reactivation of hepatitis B virus (HBV) or further dissemination of TB. Transaminase levels are normal in inactive CHB infection and become higher than normal during reactivation [12]. Diagnosing DILI requires obtaining a history of recent drug exposures, ruling out other potential causes, and acquiring evidence of improvement of liver injury upon drug discontinuation or recurrence with restarting the drug [14]. Anti-tubercular therapy (ATT) such as rifampin, isoniazid, and pyrazinamide commonly causes DILI, except for ethambutol. Rifampin can occasionally cause hepatocellular injury but usually causes cholestatic injury and unconjugated hyperbilirubinemia via interference with bilirubin uptake. Isoniazid and pyrazinamide usually cause hepatocellular injury [15].

His clinical presentation raised concerns for disseminated TB which prompted the initiation of RIPE while awaiting culture. The positive QuantiFeron-TB Gold test, positive AFB stain of the ileal tissue, and evidence of miliary TB on CT imaging suggested disseminated TB. Commonly, ATT duration is six to nine months for pulmonary TB. This treatment duration is generally applied to disseminated TB and extrapulmonary TB, except for cases of the central nervous system (CNS) or bone involvement, in which nine to twelve months is suggested [3,16,17]. A cardiac tuberculoma can achieve almost complete regression with standard treatment duration and surgery may not be necessary [12].

Administration of RIPE was complicated by severe thrombocytopenia with bone marrow biopsy suggesting a medication effect likely from rifampin-induced immune thrombocytopenia [18]. The patient completed two weeks of RIPE before transitioning to therapy with levofloxacin, amikacin, Solu-Medrol, and entecavir. Levofloxacin and amikacin were held after approximately one week of treatment due to possible adverse drug reactions (worsening transaminitis) and paradoxical reaction (clinical worsening of TB following initial improvement while on anti-TB treatment) [19]. RIPE was restarted but severe thrombocytopenia reoccurred. Multiple platelet transfusions, IVIG therapy, and rifampin cessation ultimately lead to a recovery of platelets. Eventually, the patient was discharged with entecavir, prednisone, isoniazid, pyrazinamide, ethambutol, and levofloxacin. Difficulties with diagnosis, adverse effects from treatments, and issues with communication ultimately prolonged the patient's course to nearly two and a half months.

## Conclusions

In summary, intestinal TB can present very similarly to inflammatory bowel disease (IBD); however, their treatments are diametrically opposed. Misdiagnosis as IBD could have led to the administration of immunosuppressants, which without concomitant TB treatment could have proven to be fatal. Effective use of appropriate translation resources and a systematic approach to clinical investigation can lead to early diagnosis of disseminated TB and prevention of the initial misdiagnosis of IBD. 
